# Cobalt nanoparticles as novel nanotherapeutics against *Acanthamoeba castellanii*

**DOI:** 10.1186/s13071-019-3528-2

**Published:** 2019-06-03

**Authors:** Ayaz Anwar, Arshid Numan, Ruqaiyyah Siddiqui, Mohammad Khalid, Naveed Ahmed Khan

**Affiliations:** 1 Department of Biological Sciences, School of Science and Technology, Sunway University, 47500 Subang Jaya, Selangor Malaysia; 2 Graphene and Advanced 2D Materials Research Group, School of Science and Technology, Sunway University, 47500 Subang Jaya, Selangor Malaysia

**Keywords:** *Acanthamoeba*, Cyst, Cobalt nanoparticles, Antimicrobial

## Abstract

**Background:**

Species of *Acanthamoeba* are facultative pathogens which can cause sight threatening *Acanthamoeba* keratitis and a rare but deadly brain infection, granulomatous amoebic encephalitis. Due to conversion of *Acanthamoeba* trophozoites to resistant cyst stage, most drugs are found to be ineffective at preventing recurrence of infection. This study was designed to test the antiacanthamoebic effects of different cobalt nanoparticles (CoNPs) against trophozoites and cysts, as well as parasite-mediated host cell cytotoxicity.

**Methods:**

Three different varieties of CoNPs were synthesized by utilizing hydrothermal and ultrasonication methods and were thoroughly characterized by X-ray diffraction and field emission scanning electron microscopy. Amoebicidal, encystation, excystation, and host cell cytopathogenicity assays were conducted to study the antiacanthamoebic effects of CoNPs.

**Results:**

The results of the antimicrobial evaluation revealed that cobalt phosphate Co_3_(PO_4_)_2_ hexagonal microflakes, and 100 nm large cobalt hydroxide (Co(OH)_2_) nanoflakes showed potent amoebicidal activity at 100 and 10 µg/ml against *Acanthamoeba castellanii* as compared to granular cobalt oxide (Co_3_O_4_) of size 35–40 nm. Furthermore, encystation and excystation assays also showed consistent inhibition at 100 µg/ml. CoNPs also inhibited amoebae-mediated host cell cytotoxicity as determined by lactate dehydrogenase release without causing significant damage to human cells when treated alone.

**Conclusions:**

To our knowledge, these findings determined, for the first time, the effects of composition, size and morphology of CoNPs against *A. castellanii*. Co_3_(PO_4_)_2_ hexagonal microflakes showed the most promising antiamoebic effects as compared to Co(OH)_2_ nanoflakes and granular Co_3_O_4_. The results reported in the present study hold potential for the development of antiamoebic nanomedicine.

## Background

*Acanthamoeba castellanii* belonging to the T4 genotype are causative agent of *Acanthamoeba* keratitis and granulomatous amoebic encephalitis [1]. Being free-living amoeba, *A. castellanii* is widely distributed in the environment in either of the two stages of its life-cycle: reproductive trophozoite and dormant cyst [2]. Although the diseases associated with *A. castellanii* are rare, they pose challenges in drug development, mainly due to the resistance of the cyst against therapy [3]. The high mortality rate and severe morbidity associated with *Acanthamoeba* infections is further worsened by absence of effective compounds [4]. Hence, the development of therapeutic agents that can traverse the biological barriers and have minimal toxicity to human cells is needed.

Nanoparticles have shown promise in the antimicrobial applications against vast diversity of microorganisms including bacteria, fungi, viruses and parasites [5–7]. Their small size and high surface area make them ideal candidates for drug delivery [8]. Silver, gold, copper and iron oxide nanoparticles have been widely used as potent therapeutic agents against bacteria due to their possible interaction with DNA, and inherent antibacterial properties [9–11]. However, at present there are only a few reports for the use of nanoparticles against free-living amoebae. Among metal nanoparticles, gold and silver conjugated with different drugs and natural compounds have been found effective against *A. castellanii* [12–16]. However, the effects of unconjugated metal nanoparticles against *A. castellanii* have not been studied extensively. In recent reports, titanium oxide nanoparticles have shown *in vitro* antiacanthamoebic potential triggered by ultraviolet radiation [17], while doping with zinc oxide nanoparticles has shown improved photochemotherapy [18]. In another report, gold and silver nanoparticles have been shown to improve the antiacanthamoebic effects of commercially available contact lens disinfection solutions [19]. Previous studies with metal ions showed that *A. castellanii* was unaffected by moderately high levels of Cu and Zn but was sensitive to the presence of Pb and Hg ions [20]. Cobalt is an essential element, present in vitamin B_12_ which regulates the metabolism of fatty acids and also plays a very important role in forming amino acids and proteins in nerve cells [21]. Cobalt compounds also have medical and medicinal values in the biomedical implants and treatment of anemia, respectively [22, 23]. CoNPs have gained significant importance for applications in biomedicine, catalysis and as antibacterials [24]. CoNPs are found to be biosafe and have been shown to have minimal hemolytic activity [24]. Several studies of their antimicrobial activity have been reported against bacteria and fungi [25, 26]. CoNPs and complexes containing cobalt are also reported to be active against parasites. For example, Marimuthu et al. [27] reported biologically synthesized CoNPs to be effective against malaria and dengue vectors. In another report, a cobalt complex of lapachol has shown to inhibit the growth of *A. castellanii* [28]. However, to the best of our knowledge, the effects of CoNPs against free-living amoebae have not been determined.

In the present study, we report for the first time the antiacanthamoebic activity of CoNPs against a clinical isolate belonging to the T4 genotype. Three different compositions of cobalt nanomaterials were synthesized using hydrothermal and ultrasonication methods including Co_3_O_4_ nanograins, Co_3_(PO_4_)_2_ microflakes and Co(OH)_2_ nanoflakes. The effects of CoNPs composition, morphology and size were studied against *A. castellanii* by using amoebicidal, host cell cytopathogenicity, encystation and excystation assays. Furthermore, the cytotoxicity of CoNPs was also evaluated to confirm the biosafety of these materials. Considering the challenges in developing effective new drugs against neglected diseases, it is hoped that readily synthesized CoNPs can be a useful alternative in targeting infections caused by *A. castellanii*.

## Methods

### Chemicals

Cobalt chloride hexahydrate (CoCl_2_.6H_2_O), cobalt acetate tetrahydrate [Co(CH_3_COO)_2_.4H_2_O)] and di-sodium hydrogen phosphate (Na_2_HPO_4_) anhydrous were purchased from Sigma-Aldrich (San Francisco, USA). Ammonia water (NH_4_OH 28%) and CH_4_N_2_O were purchased from Merck (Darmstadt, Germany). In-house prepared deionized (DI) water was used throughout the experiment.

### Synthesis

#### Synthesis of Co_3_O_4_ nanograins

The Co_3_O_4_ nanograins were prepared by simple hydrothermal reaction. The process was as follows: 1 mM of CoCl_2_·6H_2_O was dissolved in 20 ml of DI water and 15 ml of 6% NH_4_OH was added drop-wise at a rate of 1 ml/min followed by 1 h of constant stirring at room temperature. Finally, the total 50 ml mixture was then transferred into a 100 ml Teflon-lined stainless-steel autoclave and the hydrothermal reaction was performed at 150 °C for 5 h. The precipitates formed during the hydrothermal reaction were collected, washed several times with an excess of DI water and ethanol by centrifugation, and dried at 60 °C in a hot air oven.

#### Synthesis of Co(OH)_2_ nanoflakes

The Co(OH)_2_ nanoflakes were also prepared by hydrothermal reaction. A total of 1 mmol Co(CH_3_COO)_2_·4H_2_O was dissolved in 10 ml of DI water under constant stirring. Then, 13 ml of 6% NH_4_OH was added into the reaction mixture, stirred for 1 h, and then 2 ml of N_2_H_4_ solution was added into above mixture. The total volume of above mixture was raised to 75 ml by adding 50 ml of DI water. Subsequently, the mixture (75 ml) was transferred into a 100 ml Teflon-lined stainless-steel autoclave and the hydrothermal reaction was performed at 180 °C for 12 h. Finally, the collected precipitation was washed with DI water and ethanol and dried in the oven at 60 °C.

#### Synthesis of Co_3_(PO_4_)_2_

In order to prepare Co_3_(PO_4_)_2_, CoCl_2_·6H_2_O was dissolved in 40 ml of DI water under constant stirring. A 10 ml solution containing 1 mmol of N_2_HPO_4_ was added in above solution under constant stirring. The whole solution mixture was then subjected to ultrasonication using a horn sonicator for 30 min. The colloidal solution formed during sonication was washed with copious amounts of DI water by centrifugation and dried in a hot air oven at 60 °C. Finally, the dried powder was crushed using an agate mortar and pestle calcined at 300 °C for 3 h in a muffle furnace. The changing color for powder from pink to dark blue confirmed the successful formation of Co_3_(PO_4_)_2_.

### Characterization techniques

The surface morphological studies were performed using a JEOL JSM-7600F field emission scanning electron microscope (FESEM). The phase identification and crystallinity of the prepared materials were obtained using a Philips X’pert X-ray diffractometer (XRD) using Cu-K*α* X-ray radiation (λ = 1.5418 Ǻ) at a rate of 0.02 s^−1^ for the 2*θ* range of 15 to 75 degrees.

### Cultures of *A. castellanii*

A clinical isolate of *A. castellanii* (ATCC 50492) belonging to the T4 genotype was cultured in 10 ml of growth medium consisting of 0.75% (w/v) proteose peptone, 0.75% (w/v) yeast extract and 1.5% (w/v) glucose (PYG) in 75-cm^2^ tissue culture flasks at 30 °C. The flask reached confluency in around 48 h.

### Amoebicidal assays

Amoebicidal assays were carried out as described previously [13, 29]. Briefly, active adherent trophozoites were obtained by changing PYG with RPMI-1640 and placing the flask on ice for 15 min followed by 5 min of gentle tapping to detach the trophozoites. This suspension was centrifuged for 10 min at 3000×*g* and the obtained pellet was resuspended in 1 ml of RPMI-1640. The population of *A. castellanii* was determined by enumerating cells using a hemocytometer. The amoebicidal effects of CoNPs were determined by treating 5 × 10^5^ *A. castellanii* trophozoites with various concentrations ranging from 10 to 100 µg/ml in RPMI-1640 in 24-well plates. Chlorhexidine was used as a positive control in the assay and untreated amoebae in RPMI-1640 alone as a negative control. Plates were incubated for 24 h at 30 °C. Next, the viability was determined by Trypan blue exclusion assay. Trypan blue (0.1%) was added to each well and unstained cells (viable) were counted using a hemocytometer.

### Encystation assays

Encystation assays were performed as previously described [14, 30]. A total of 5 × 10^5^ *A. castellanii* trophozoites were incubated with CoNPs in a 24-well plate in PBS containing an encystation medium consisting of 5 mM MgCl_2_ and 8% glucose. The plates were incubated at 30 °C for 72 h, followed by adding 0.25% sodium dodecyl sulfate (SDS) to solubilize trophozoites but not mature cysts. Finally, the remaining cysts were counted using a hemocytometer.

### Excystation assays

*Acanthamoeba castellanii* cysts were prepared as described previously [31]. Briefly, 1 × 10^6^ trophozoites were inoculated on non-nutrient agar plates and the plates were incubated at 30 °C for up to 14 days. Next, the cysts were scraped with 5 ml of phosphate-buffered saline (PBS) and centrifuged at 3000×*g* for 10 min to collect the cysts pellet. These cysts were then resuspended in 1 ml of PBS, enumerated using a hemocytometer, and kept at 4 °C for use in excystation assays. The effects of CoNPs on excystation were measured by incubating 1 × 10^5^ *A. castellanii* cysts with nanoparticles in 24-well plates at 30 °C and observed under an inverted microscope every 24 h for up to 72 h to detect the emergence of trophozoites. The trophozoites emerged were counted using a hemocytometer.

### HaCaT cell culture

Human keratinocyte cells were cultured in RPMI-1640 supplemented with 10% fetal bovine serum (FBS), 1% (2 mM) l-glutamine, 1% penicillin-streptomycin and 1% nonessential amino acid (NEAA) as previously described [32]. The cells were incubated at 37 °C in a 5% CO_2_ incubator with 95% humidity for the formation of uniform monolayers. After the removal of existing media, confluent flasks were trypsinized with 2 ml of trypsin to detach the cells which were then centrifuged at 2000×*g* for 5 min. The resulting pellet was resuspended in 30 ml media and seeded into 96-well plates for use in cytotoxicity and cytopathogenicity assays once a uniform monolayer was formed.

### Cell cytotoxicity assays

Cytotoxicity of CoNPs towards human keratinocytes cells was tested by treating 100 µg/ml nanoparticles with HaCaT monolayers in 96-well plates by using a lactate dehydrogenase (LDH) assay as described previously [32]. The cells were incubated in a 37 °C, 5% CO_2_ incubator for 24 h. Untreated cells in RPMI-1640 only were taken as negative controls and 1% Triton X-100 was used as a positive control. Following the 24 h incubation, cell death was determined as extent of LDH release from damaged cells detected by an LDH detection kit (Invitrogen, Illinois, USA) at 490 nm. Percentage cytotoxicity was calculated as follows: (Sample absorbance − Negative control absorbance)/(Positive control absorbance − Negative control absorbance) × 100.

### *Acanthamoeba castellanii*-mediated host cell cytopathogenicity

Amoeba-mediated cells cytotoxicity was determined by LDH assay as described previously [13]. Briefly, 5 × 10^5^ *A. castellanii* trophozoites were treated with CoNPs in 24-well plates with RPMI-1640 and incubated at 30 °C for 2 h. Next, the amoebae were centrifuged at 3000×*g* for 10 min to separate extracellular components, and the pellet was resuspended in 200 µl of fresh RPMI-1640 before addition to HaCaT cell monolayers. The plates were incubated for 24 h at 37 °C in a 5% CO_2_ incubator. Negative control was consisting of cells with RPMI-1640 only and a positive control was achieved by 100% cell death by lysing cells with 1% Triton X-100. Next, the supernatant of each well was collected and the release of LDH was measured at an absorbance of 490 nm using an LDH detection kit. The percentage cytopathogenicity was calculated by formula used for determining cell cytotoxicity.

### Statistical analysis

All presented results are representatives of several experiments performed in duplicate and are denoted as the mean ± standard error. Microsoft Excel worksheets were prepared for data obtained from biological assays; for statistical significance, Student’s t-test was performed comparing test sample effects with negative control. The threshold level of significance was *P* < 0.05, using a two-sample t-test and two-tailed distribution.

## Results

### X-ray diffraction

**Fig. 1:**
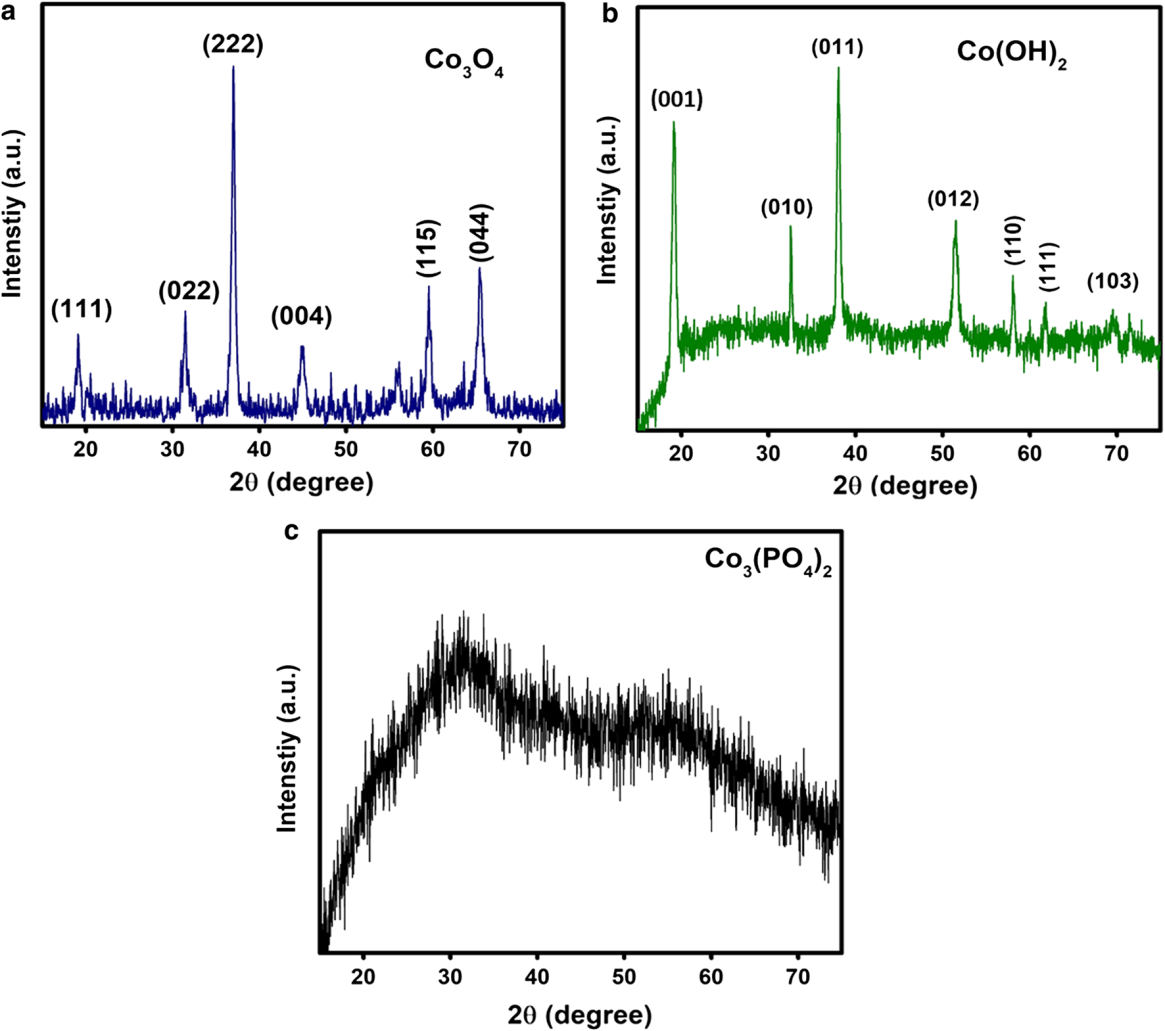
Representative X-ray diffraction patterns of **a** Co(HO)_2_ nanoflakes, **b** Co_3_O_4_ nanograins and **c** Co_3_(PO_4_)_2_

Pure Co_3_O_4_ nanograins displayed characteristic peaks at 2*θ* values of 18.81°, 30.95°, 36.47°, 44.35°, 58.72° and 64.52° that correspond to (111), (022), (222), (004), (115) and (044) lattice planes, respectively (Fig. 1a). All the diffraction peaks of Co_3_O_4_ nanograins are well indexed with the cubic phase with space group Fd-3m (ICDD-PDF 96-900-5893) [33]. The XRD pattern of Co(OH)_2_ nanoflakes showed sharp peaks at 2*θ* values of 19.1°, 32.5°, 38.0°, 51.0°, 58.1°, 61.4° and 69.7° which can be attributed to the (001), (010), (011), (012), (110), (111) and (103) lattice planes, respectively, of cobalt hydroxide (Fig. 1b). All characteristics peaks of Co(OH)_2_ nanoflakes can be indexed to the hexagonal crystal structure of Co(OH)_2_ (PDF 96-101-0268, space group P-3m1) [34]. In case of Co_3_(PO_4_)_2_, no sharp and well-defined peaks were observed due to its highly amorphous nature (Fig. 1c). This amorphous nature is due to the irregular growth of Co_3_(PO_4_)_2_ at the atomic scale during the ultrasonication. Similar results were also demonstrated by Kim et al. [35].

### Field emission scanning electron microscopy

**Fig. 2:**
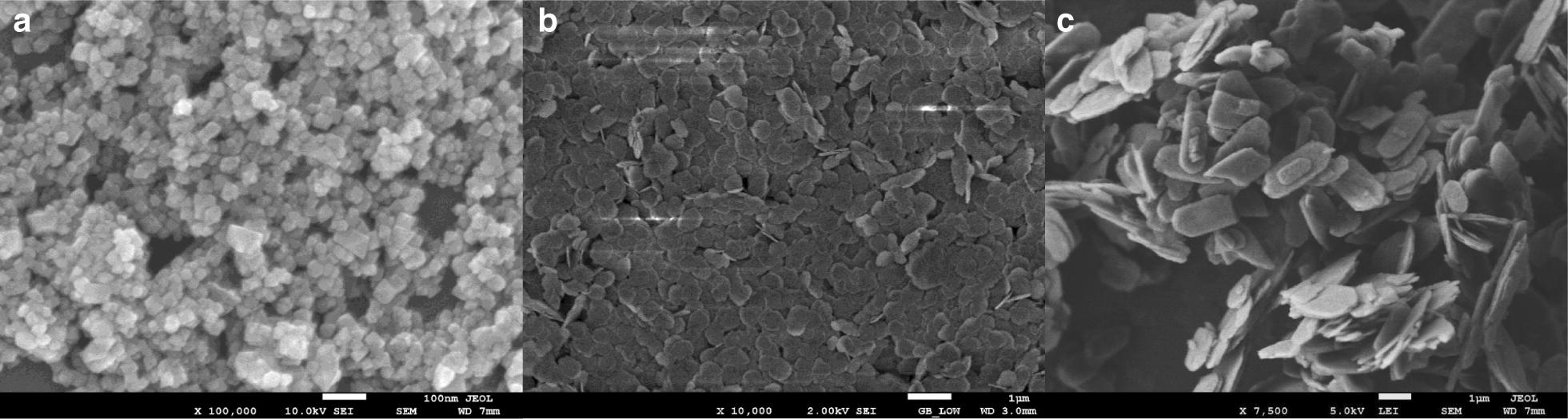
Representative FESEM images of **a** Co_3_O_4_ nanograins, **b** Co(OH)_2_ nanoflakes and **c** Co_3_(PO_4_)_2_. Scale-bars: **a** 100 nm; **b c**, 1 µm

In order to examine the surface morphology of the prepared nanostructures, FESEM was employed to take micrographs. Figure 2 shows the FESEM images of the prepared materials. Figure 2a depicts the granular morphology prepared by Co_3_O_4_ by hydrothermal synthesis. The observed particle size was around 35 to 40 nm. Figure 2b shows the flake-like structure of Co(OH)_2_ with a flake size of almost 100 nm while Fig. 2c shows the Co_3_(PO_4_)_2_ hexagonal microflakes.

### CoNPs exhibited amoebicidal properties

**Fig. 3:**
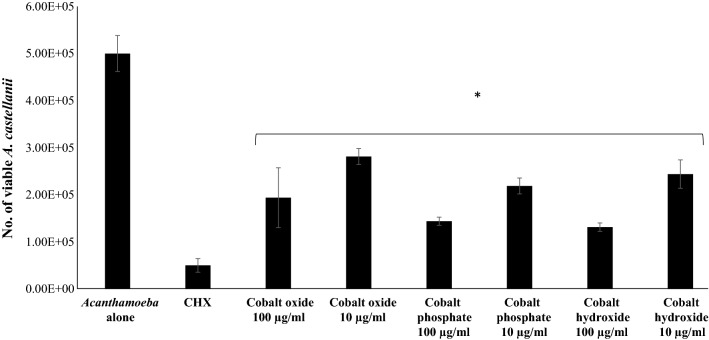
Amoebicidal effects of CoNPs on *A. castellanii* belonging to the T4 genotype. In brief, 5 × 10^5^ *A. castellanii* trophozoites were incubated with different CoNPs at 30 °C for 24 h after which viability was determined by staining with Trypan blue. The results show significant amoebicidal activity when compared to the control. *P < 0.05

Amoebicidal assays were carried out to assess the effect of size, composition and morphology of CoNPs on the viability of *A. castellanii* (Fig. 3). CoNPs were dissolved in deionized water upon sonication for 1 h, and a stock aqueous solution of 5 mg/ml was prepared to evaluate the overall antiacanthamoebic activity. Numbers of trophozoites were maintained at 5 × 10^5^ in the negative control comprising of amoeba in RPMI-1640 alone. *Acanthamoeba castellanii* trophozoites were reduced to 5 × 10^4^ when treated with 100 µg/ml of chlorhexidine used as a positive control. The viability of *A. castellanii* was significantly reduced upon challenging amoebae with CoNPs at both 10 and 100 µg/ml. A concentration of 100 µg/ml CoNPs caused around 70% reduction in the viability of *A. castellanii*, while 10 µg/ml caused around 50% reduction. Among the three compositions of CoNPs tested, Co_3_(PO_4_)_2_ exhibited most potent amoebicidal effects (Fig. 3).

### CoNPs inhibited the encystation of *A. castellanii*

**Fig. 4:**
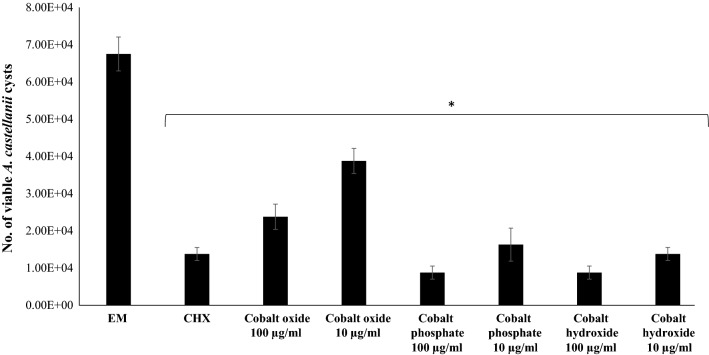
Effects of CoNPs on the encystation of *A. castellanii* belonging to the T4 genotype. In brief, 5 × 10^5^ *A. castellanii* trophozoites were incubated with an encystation media (8% glucose, 5 mM MgCl_2_) and different CoNPs at 30 °C for 72 h. Subsequently, cysts were enumerated after treating with 0.25% SDS. The results show significant inhibition of encystation when compared to the control. *P < 0.05

The encystation assays were performed to test whether CoNPs can inhibit the phenotypic differentiation of *A. castellanii* trophozoites into cysts or not. The results revealed a similar trend of effectivity as in the amoebicidal assay, and except for Co_3_O_4_ nanograins both nanoparticles showed potent anti-encystation effects at both 100 and 10 µg/ml (Fig. 4). Upon incubation of 1 × 10^5^ *A. castellanii* trophozoites with encystation medium as negative control, 6.75 × 10^4^ cysts were obtained after 72 h. Comparatively, the numbers of cysts were significantly reduced after treatment with CoNPs, especially in the case of Co_3_(PO_4_)_2_ and Co(OH)_2_ where only 8.75 × 10^3^ cysts were enumerated in each, as compared to negative control (6.75 × 10^4^).

### CoNPs inhibited excystation of *A. castellanii*

**Fig. 5:**
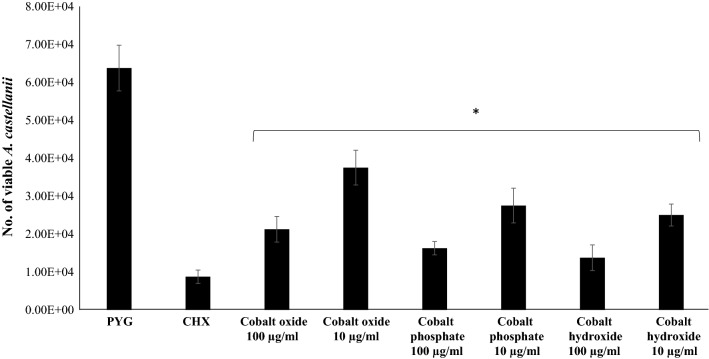
Excystation assay was performed to evaluate the differentiation of *A. castellanii* from cysts into trophozoites. Briefly, 5 × 10^5^ *A. castellanii* cysts were incubated with CoNPs in growth medium (PYG) at 30 °C for 72 h. After incubation, cysts emerged into trophozoites were enumerated using hemocytometer. The results show significant inhibition of encystation when compared to the control. *P < 0.05

The excystation assay revealed that all three compositions of CoNPs exhibited a moderate inhibition of excystation (Fig. 5). Again, the smallest amongst the three tested CoNPs, Co_3_O_4_ nanograins, showed the least effect on excystation at 10 µg/ml as compared to the negative control which was untreated cysts in PYG (Fig. 5).

### CoNPs did not cause cytotoxicity against human cells and inhibited *A. castellanii*-mediated host cell cytopathogenicity

**Fig. 6:**
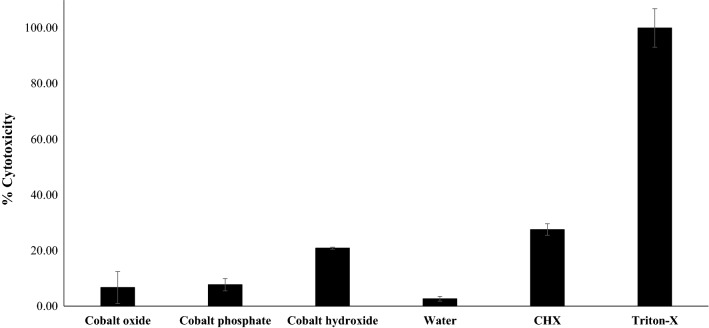
CoNPs presented limited cytotoxicity to HeLa cells. Briefly, 100 µg/ml of CoNPs were added to HeLa cells and incubated for 24 h at 37 °C in a 5% CO_2_ incubator. The result of cytotoxicity for the most potent antiamoebic CoNPs, that is Co_3_(PO_4_)_2,_ shows lower cytotoxicity when compared to 100 µg/ml of the antiamoebic drug chlorhexidine

**Fig. 7:**
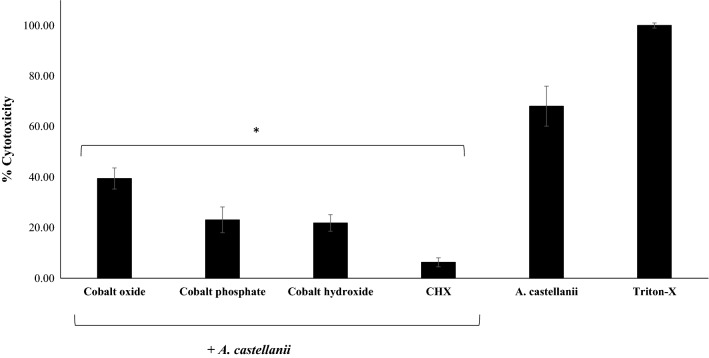
Pre-treatment of amoeba with CoNPs inhibited HeLa cell cytopathogenicity. In brief, 5 × 10^5^ *A. castellanii* trophozoites were incubated with 100 µg/ml of CoNPs at 30 °C for 2 h. Treated amoeba were then added to HeLa cells and incubated for 24 h at 37 °C in a 5% CO_2_ incubator. The results show significant inhibition of cytopathogenicity when compared to untreated amoeba. *P < 0.05

Cell cytotoxicity assays were carried out to determine the toxicity of CoNPs against HaCaT cells at the highest dose of 100 µg/ml. CoNPs produced minimal damage to human cells with 7, 8 and 21% cytotoxicity for oxide, phosphate and hydroxide, respectively (Fig. 6). These results suggest that these nanoparticles are biosafe and have the potential for further exploration against infections caused by *A. castellanii*. On the other hand, to assess the possible inhibition of amoeba’s pathogenicity to host cells, host cell cytopathogenicity assays were performed. The results revealed that pre-treatment of *A. castellanii* with CoNPs significantly reduced the host cell toxicity. Untreated *A. castellanii* produced around 70% cytotoxicity to HaCaT cells, whereas amoebae pre-treated with 100 µg/ml of Co_3_(PO_4_)_2_ and Co(OH)_2_ reduced this pathogenicity to around 20% (Fig. 7).

## Discussion

*Acanthamoeba* spp. cause diseases of devastating morbidity and mortality. The risk of *Acanthamoeba* keratitis is significantly higher in contact lens wearers. Despite the progress in eye care industry, none of the contact lens solutions are effective against *Acanthamoeba* keratitis [36]. Furthermore, granulomatous amoebic encephalitis, the brain disease caused by *A. castellanii*, almost always results in death [37]. Despite the high associated rates of mortality and morbidity, infections caused by *Acanthamoeba* are still considered as rare and neglected diseases. To date, there is no single drug available to treat infections caused by *A. castellanii* [38]. Hence, there is an urgent need for the development of novel therapeutic options that can effectively circumvent the drawbacks that current drugs possess.

Nanoparticle-drug conjugates have recently been observed to exhibit enhanced antiamoebic effects against *A. castellanii* as compared to drugs alone. In this context, a variety of drugs have been tested including chlorhexidine, amphotericin B, nystatin, fluconazole, phenytoin, phenobarbitone, diazepam, etc. [12–15]. However, the effects of unconjugated metallic nanoparticles have scarcely been studied against *A. castellanii* [18, 19]. In this study, we discovered the antiacanthamoebic effects of CoNPs. Three compositions of CoNPs were successfully synthesized using different synthetic techniques including hydrothermal and ultrasonication methods. Nanomaterials were thoroughly characterized by using X-ray diffraction and field emission scanning electron microscopy before biological studies. The different varieties of CoNPs were tested in order to evaluate the effect of composition, morphology and size of nanoparticles with respect to their antiamoebic effects. This strategy was taken to optimize the best antiacanthamoebic CoNPs. Amoebicidal assay results revealed that flake-shaped CoNPs inhibited the viability of *A. castellanii* more effectively than granular particles. Furthermore, these CoNPs were also tested for cytotoxicity against normal human cells by LDH cytotoxicity assays to evaluate their biosafety. Moreover, CoNPs also significantly inhibited the encystation and excystation which is an essential requirement for drug development against *A. castellanii* along with their potency to cross blood-brain barrier. It is also interesting to note that the CoNPs used in this study were non-conjugates (free of any drugs or compounds), as compared to the previously reported nanoparticle-drug conjugates. However, these nanoparticles exhibited comparable antiamoebic effects (killing more than 70% of amoebae) *versus* antiamoebic nanoparticle-drug conjugates with chlorhexidine [12] and amphotericin B [13], but only at higher concentration of 100 µg/ml.

Regarding the bactericidal activity of CoNPs, the literature suggests that CoNPs can diffuse inside the cell interacting with the cell membrane, producing oxidative stress and finally causing DNA damage [39]. However, currently there is no knowledge of any associated mechanism of CoNPs against parasites. Khalil et al. [40], showed that CoNPs displayed antileishmanial activity against both of the axenic promastigote and amastigote cultures, but the mechanism of inhibition was not described [40]. In addition to applications utilizing their bioactivity, they have also been used to isolate microbes from biological matrix due to their magnetic nature. Heli et al. [41], designed cobalt-zinc ferrite quantum dots for the detection of *Leishmania major*.

## Conclusions

These findings present the potential use of three different kinds of CoNPs against *A. castellanii* due to their significant amoebicidal effects and inhibition of encystation and excystation. The smallest sized granular Co_2_O_3_ nanoparticles showed minimum antiamoebic effects as compared to flakes of Co_3_(PO_4_)_2_ and Co(OH)_2_ which exhibited better overall effects. These CoNPs were also found to reduce the host cell cytotoxicity caused by *A. castellanii*, while causing minimal toxicity to normal cells. However, further studies are required to ascertain the complete molecular mechanism of action which at this stage was beyond the scope of the present study. The findings from present study have established the potential *in vitro* utility of CoNPs with tunable composition and morphology against *A. castellanii*. Chlorhexidine, pentamidine and miltefosine are promising drugs currently used for treatment, but the most important shortcoming associated with these drugs is their high host cell toxicity. Besides this limitation, the growth of drug resistance is a ticking time bomb in pathogens, especially against these older organic drug molecules; this needs to be addressed by finding therapeutics of alternative translational values. These materials are easy to synthesize with low cost and high yields, and their possible impurities are significantly less as compared to organic synthetic protocols and require far less expertise as compared to dedicated and sophisticated organic drug synthesis. Since a lack of progress in the development of new and effective drug molecules has majorly contributed to the high mortality and morbidity of parasitic infectious diseases, it is anticipated that these CoNPs could serve as a novel alternative against *A. castellanii*. Nevertheless, mechanistic and *in vivo* studies are required before recommending the translational value of these CoNPs.

## Data Availability

All relevant data are provided in the manuscript and any additional data will be provided upon request.

## Abbreviations

AK: *Acanthamoeba* keratitis
GAE: granulomatous amoebic encephalitis
CoNPs: cobalt nanoparticles
DI: deionized
FESEM: field emission scanning electron microscope
XRD: X-ray diffraction
PYG: proteose peptone, 0.75% (w/v) yeast extract and 1.5% (w/v) glucose
RPMI: Roswell park memorial institute
PBS: phosphate saline buffer
SDS: sodium dodecyl sulfate
FBS: fetal bovine serum
NEAA: nonessential amino acid
LDH: lactate dehydrogenase
ICDD: the international centre for diffraction data
PDF: powder diffraction file
